# Annotations

**Published:** 1888-06-16

**Authors:** 


### Annotations.

**Sympathy and Congratulation.** All philanthropists and those who value good works will sympathise with Mrs. Ernest Hart under the singularly harsh and uncalled for libel she has been subjected to, and will warmly endorse our congratulations upon the handsome apology she has extracted from Mr. Sinclair. Some months ago we were angered by the inquiry as to the *bonâ fides* of the promoters of the Donegal Fund. Our surprise at the suggestion of misappropriation was only exceeded by our disgust at finding that the noble self-denial displayed by Mrs. Ernest Hart in the cause of the Donegal peasants should be so ill requited. A court of law has now caused Mr. Sinclair to withdraw "fully all imputations upon Mrs. Ernest Hart, and all suggestions that she was influenced by any corrupt or indirect motions, and all imputations upon the character of the work carried on by her, and on the genuine character of the goods, the working and sale of which she has superintended and promoted, or of the technical instructions which she has been conducting." The *Pall Mall Gazette* truly declares "any harm Mr. Sinclair's statements may have caused should now be undone, and Mrs. Hart's noble and disinterested work be vindicated before all the world." Quite so, but how can the public give some enduring recognition to this feeling? By making Mrs. Hart's work a complete success! For this purpose a sum of £5,000 should be raised and presented to her for the promotion of the Donegal Industrial Fund, the work of which is at present hampered and restricted for the want of additional capital. Five thousand pounds would be ample, we believe, for all the purposes of Mrs. Hart's scheme, and if any of our readers share our views and are willing to subscribe or to assist in raising such a testimonial fund as is here suggested, we should be glad to hear from them at once. Meanwhile, anyone interested should take an opportunity of visiting and inspecting the work done by the Donegal peasants, as exhibited at 43, Wigmore Street, W.

**The Care of Consumptives.** Phthisis, or as it is popularly called, consumption, is a specially British disease. The humid climate that marks our islands, what gives our fields their unsurpassable green, and our maidens' cheeks their unrivalled roses, is peculiarly favourable to the development of diseases of the respiratory organs, and every autumn sees a flight of exiles to lands whose atmosphere is less trying than ours to their weakly lungs. The popular name, consumption, expresses well the idea we all have of this subtle disease—an inward fire, insidiously increasing its strength day by day, and destroying remorselessly youth, beauty, genius. It is often our best and fairest that consumption attacks, or those whom we hold most dear, and those from whose learning and talent we profit most. When Louis Stevenson sends a romance from his Californian refuge, or John Addington Symonds writes for us, in his shelter at Davos Platz, one of those studies of the Renaissance or of the pre-Shakespearean dramatists of whom no one knows so much as he, we study their work with gratitude not unmingled with fear, lest the strong and beautiful intellect should never again be able to offer us such gifts. These are the few, but what of the many—girls passing away in the bloom of youth, men dying in the flush of manhood, full of hope and ambition. And what of the poor—those who through the long months, the years it may be, of increasing feebleness are destitute of all the comforts that make life endurable? Where are they to go till the release comes? Their little crowded homes in narrow streets and slums are a continual torture to the irritable sensitiveness of consumptive patients, and the hospitals, as a rule, will not take them in. In such circumstances a hospital for consumption is a great boon, as the report of the Consumption Hospital at Brompton shows. The stay of the patients there averaged 70 days, or nearly three times as long as in any other hospital. Although the closing of a portion of the building for necessary repairs has made the number of in-patients smaller than usual, they amounted in all to 1,533, of whom 1,200 were discharged greatly benefited. In the out-patient department 72,170 attendances had been registered, and much good had been done. Even though consumption is, strictly speaking, incurable, much can be done in the early stages of it to arrest, and in the later, to delay its progress, and an institution which, like the hospital at Brompton, undertakes to do this, is deserving of the liberal and loyal support of the public.

**The Case for Hanging.** "The worst use you can put a man to is to hang him," was said long ago, and "the best thing you can do with a man is to electrify him," has for some time been the motto of the sensational journalist. Now there is an attempt being made to bring the two uses together; and when public opinion, as represented by a jury, has decided that a man must be put to that worst use of death, to plead that this end may be attained by electricity. It is a plea for mercy. If it be admitted that for the safety of society, as a whole, it is necessary for any one member of it to be killed, let the killing be done without inflicting unnecessary pain. Our desire is only that he may be prevented from injuring his fellow-men, and his death alone will assure us of this; but we do not want to make him suffer more than is inevitable in passing from life into death. This is the humanitarian sentiment, but the action based on it goes far beyond. Compared with the pains of ordinary natural death, hanging is easy, death by electricity luxurious. It is, so far as can be ascertained, absolutely painless, besides being instantaneous. To those who are not in love with life—and they are not few—this electric euthanasia is even desirable. Whether or not capital punishment is justifiable at all is a point we do not propose discussing; it is a question for the most thoughtful and conscientious consideration of our law-makers, and may for the present be left to them. But its justification lies not merely in the fact that thereby we permanently get rid of a dangerous criminal, but in the deterrent effect the manner of his death will have on others who might be disposed to similar crimes. Hanging is universally looked upon as degrading, and though our advancing humanity has annihilated the "privilege" of the block, some criminals are permitted to quit the world by the more "honourable" means of a bullet. Absurd and pitiful as such preferences seem in face of the terrible fact of death, they exist, and their existence cannot be ignored in settling any point in which human prejudice as well as human life is concerned. Would not such a preference exist more strongly still in favour of such a "fancy death" as the euthanasia of electricity. There are scores of rash, ignorant rather than unprincipled, adolescents, afflicted only with the immaturity and egoism of youth, who, when jilted by their sweethearts, or punished by their masters, meditate a sanguinary revenge and are deterred only by the shame of hanging, not by the fear of death. That shame keeps them from crime, and in the end they outgrow their dangerous propensities, and develop into respectable, even God-fearing citizens; but would they be so likely to do so if the death they risked lay concealed in the electric chain? "No man dieth to himself," especially he who dies a judicial death, and the best plea we can make for the utmost penalty of the law is that the fear of it may keep others from deserving it; and to this end it is well that, so far as a decent regard for mercy to the condemned criminal permits, it should be conducted in such a manner as to restrain the passions of those who, though tempted, are still innocent, by a wholesome fear of shame. It is true that death by electricity would at last, as being the means by which criminals are executed, become disparaged in public estimation; but this would be a matter of time, and in face of the benefits electricity is daily conferring on us it would be long before such a consummation was reached, longer perhaps than with any other method of execution. For such an act as taking away the life of our brethren, moreover, any simple means not absolutely cruel *per se* are the best—the simpler the better; tampering with the beneficent secrets of science is almost unjustifiable.

